# Using Affective Cognition to Enhance Precision Psychiatry

**DOI:** 10.3389/fpsyt.2018.00288

**Published:** 2018-06-29

**Authors:** Jack Cotter, Jennifer H. Barnett

**Affiliations:** ^1^Cambridge Cognition, Cambridge, United Kingdom; ^2^Department of Psychiatry, University of Cambridge, Cambridge, United Kingdom

**Keywords:** affective cognition, cognitive bias, emotion, mood disorder, neuropsychological assessment, precision medicine

Major depressive disorder affects hundreds of millions of people, is among the leading causes of disability worldwide, and is a major contributor to the overall global burden of disease (1). While a range of effective drugs are available (2), the majority of patients do not achieve symptomatic remission from their first antidepressant treatment, though it typically takes weeks or months to establish this non-responsiveness (3). This delay in the initiation of effective treatment is likely to have a detrimental effect on patient welfare and quality of life, and account for significant financial burden in the form of lost productivity, clinical costs, and welfare expenditure (4). Given the scale of this problem, even minor improvements to current practice could have a substantial personal and societal impact. Similar issues are also faced in other mental health disorders, leading many clinicians and researchers to consider novel ways to help stratify patients based on biological, psychological, and lifestyle factors that builds on standard psychiatric diagnoses. Widely termed “precision psychiatry,” this ambitious and personalized approach to medicine offers a range of exciting possibilities for optimizing effective prevention, diagnosis, and treatment of psychiatric disorders (5).

Concepts at the heart of precision psychiatry include the ability to: (i) disentangle subgroups within and across broader psychiatric diagnoses on the basis of common biological and/or psychological traits; (ii) identify individuals most likely to benefit from a particular therapeutic intervention; (iii) determine as early as possible that an intervention is ineffective in order to facilitate initiation of successful treatment; (iv) recognize early indicators of vulnerability, onset or relapse to promote early intervention. Much of the current focus of research in this area has been based on genetic and imaging data, however, there is an increasing literature suggesting that cognitive testing for affective biases may provide a brief, flexible, and non-invasive biomarker with the potential to help optimize these processes for patients with mood disorders.

There is a wealth of literature demonstrating that deficits in “cold” (emotion-independent) cognition are common across psychiatric disorders and are an important predictor of functional decline. These deficits are largely distinct from symptom severity and persist even among individuals in clinical remission (6). However, there is a growing recognition of the importance and potential clinical utility of cognitive tasks with an affective component. While deficits in the ability to accurately identify emotional facial expressions are found transdiagnostically and likely reflect more generalized neurological abnormality (7), there is evidence that measures sensitive to cognitive biases in the processing of affective information may provide a biomarker for clinical symptom expression specific to mood disorders. These are widely referred to as “hot” (emotion-laden) cognitive processes and include measures of perceptual and attentional biases, reward processing, and feedback sensitivity. To put this into context, patients with major depressive disorder have been reported to display a range of mood-congruent processing biases, responding slower, and less accurately to positively valenced stimuli, exhibiting heightened sensitivity to negative feedback and a greater tendency to interpret ambiguous stimuli (such as faces) negatively compared to non-depressed controls (8).

Recent models of mood disorders have suggested that these cognitive biases may be a core feature of the illness, which arise as a result of abnormalities in underlying neurotransmitter systems and play a causal role in the onset and maintenance of these conditions (9). In contrast to traditional “cold” cognitive tasks, evidence has demonstrated that hot cognitive measures are sensitive to clinical treatment. Importantly, the normalizing of these affective processing abnormalities (based both on behavioral and imaging data) has been reported to predict later clinical response, providing an early indicator of treatment efficacy prior to observable symptomatic improvement (10, 11). These changes have been detected early in the treatment course, even after a single dose of an antidepressant, suggesting these tasks may be able to play a key role in optimizing successful treatment initiation (12).

Vital to the advance of such knowledge is the translation from robust research finding in to a clinically useful tool. This is an area where great strides are being made for the first time, with efforts currently underway to develop testing platforms and algorithms that can easily be used and interpreted by clinicians. This includes a major multinational, randomized, controlled trial investigating the clinical and cost-effectiveness of a medical device incorporating affective cognitive assessment to guide antidepressant treatment selection in patients with major depressive disorder (13). Though it has yet to be robustly explored, monitoring of changes in affective biases may also be particularly relevant for at-risk groups, for whom the ability to identify early signs of onset or relapse before the presence of full-blown symptoms may help facilitate early intervention.

An important consideration is that not all patients with depression exhibit these cognitive biases, which may help to provide insights into different etiologies underlying the disorder. This may also allow us to identify a subgroup of patients for whom particular drugs or psychological therapies may be more effective. For example, “cognitive restructuring” is a core aspect of cognitive behavioral therapy, which attempts to help patients identify and challenge many of these dysfunctional cognitive biases. Screening for such biases may have the potential to help optimize treatment. There is also emerging evidence that these biases may themselves provide a direct target for novel therapeutic interventions. Penton-Voak et al. provide a detailed review of this literature and have developed a cognitive training task that uses feedback in an effort to modify maladaptive affective biases using facial stimuli (14). Preliminary evidence indicates that this change in bias is associated with improvements in mood, further supporting the proposed causal relationship between these factors and paving the way for the development of behavioral interventions that can be delivered largely autonomously by patients at home as an adjunct to standard clinical treatment.

While much of this work still remains at an early stage in the translational process, preliminary evidence suggests this is a promising area with potentially diverse clinical applications. Though no single biomarker is likely to address all of the issues mentioned at the start of this article, we believe continued advances in the characterization and operationalization of affective cognitive processes have the potential to compliment ongoing multidisciplinary research seeking to enhance precision psychiatry and improve the lives of patients worldwide.

## Author contributions

JC drafted and JB critically reviewed the manuscript. Both authors approved the final manuscript for submission for publication.

### Conflict of interest statement

The authors are employees of Cambridge Cognition Ltd. The authors declare that the research was conducted in the absence of any commercial or financial relationships that could be construed as a potential conflict of interest.
